# An Improved Yaw Estimation Algorithm for Land Vehicles Using MARG Sensors

**DOI:** 10.3390/s18103251

**Published:** 2018-09-27

**Authors:** Gang Shi, Xisheng Li, Zhengfu Jiang

**Affiliations:** 1 School of Automation and Electrical Engineering, University of Science and Technology Beijing, Beijing 100083, China; shigang_upc@163.com (G.S.); jzf_ustb@163.com (Z.J.); 2 College of Information and Control Engineering, China University of Petroleum, Qingdao 266580, China; 3 Shengli College, China University of Petroleum, Dongying 257061, China; 4 Beijing Engineering Research Center of Industrial Spectrum Imaging, Beijing 10083, China

**Keywords:** attitude estimation, Kalman filter, land vehicle, magnetic angular rate and gravity (MARG) sensor, quaternion, yaw estimation

## Abstract

This paper presents a linear Kalman filter for yaw estimation of land vehicles using magnetic angular rate and gravity (MARG) sensors. A gyroscope measurement update depending on the vehicle status and constraining yaw estimation is introduced. To determine the vehicle status, the correlations between outputs from different sensors are analyzed based on the vehicle kinematic model and Coriolis theorem, and a vehicle status marker is constructed. In addition, a two-step measurement update method is designed. The method treats the magnetometer measurement update separately after the other updates and eliminates its impact on attitude estimation. The performances of the proposed algorithm are tested in experiments and the results show that: the introduced measurement update is an effective supplement to the magnetometer measurement update in magnetically disturbed environments; the two-step measurement update method makes attitude estimation immune to errors induced by magnetometer measurement update, and the proposed algorithm provides more reliable yaw estimation for land vehicles than the conventional algorithm.

## 1. Introduction

As a set of Euler angles, the yaw, pitch, and roll represent the orientation of a body frame with respect to a reference frame. The pitch and roll are also referred to as attitude. Yaw and attitude estimation are widely used in vehicular technologies including driver assistance [1,2,3], vehicle safety [4,5], etc. In recent years, magnetic angular rate and gravity (MARG) sensors [6] are widely used in orientation estimation. A MARG sensor consists of a triaxis magnetometer, a triaxis gyroscope, and a triaxis accelerometer. Reasonable installation and calibration make it acceptable to assume that the sensor frames are aligned with the body frame. Hence, a MARG sensor can measure the geomagnetic field, angular rate of the body frame, and the gravity resolved in the body frame in undisturbed environments.

In order to obtain an orientation estimation of the body frame, we can integrate the gyroscope output based on an initial value, but the result will drift away with time because of gyroscope measurement errors [7]. Alternatively, we can solve the Wahba problem [8] using magnetometer and accelerometer outputs, but the sensor outputs are apt to be interfered by motion accelerations and magnetic disturbances [9,10]. Therefore, to make the most of the information from MARG sensors and obtain robust orientation estimation, many fusion algorithms have been studied. These algorithms can be classified into two categories: one is based on complementary filters, which realize the fusion in frequency domain [11,12,13], and the other is based on Kalman filters, which employ a stochastic approach [14,15,16]. Kalman filter based algorithms consist of two basic processing, i.e., time propagation and measurement update. In time propagation, the gyroscope output is used to predict the orientation and the result is called a priori estimation; in measurement update, the magnetometer and accelerometer outputs are used to correct the a priori estimation and the result is called a posteriori estimation.

This paper studies on the yaw estimation of land vehicles using MARG sensors and Kalman filter. Our work is based on the orientation estimation because the yaw can be extracted from orientation estimation; at the same time, we focus on special problems in yaw estimation.

Many algorithms of orientation estimation have been studied. They are different in terms of the state vector, filter structure, etc., but they are the same at one point, i.e., the magnetometer output is used to correct the yaw estimation. In fact, the correction of yaw estimation is implemented only by magnetometer measurement update because the accelerometer output only provides attitude information. Hence, one problem in yaw estimation is that it is vulnerable to magnetic disturbances. Magnetic disturbances include hard iron effects, soft iron effects, and environmental magnetic disturbances [17]. The hard and soft iron effects can be compensated through magnetometer calibration, but the environmental magnetic disturbances cannot be effectively compensated because of their nondeterminacy [18,19,20]. In the following, the “magnetic disturbances” refers specifically to the environmental magnetic disturbances.

Methods to handle magnetic disturbances have been proposed. A measurement vector selection scheme based on norm comparison was designed in [7]. Costanzi et al. [21] scaled down the gain associated with the magnetometer output when two particular angular constraints are violated. Wu et al. [22] did not use the magnetometer output if its norm was too big or too small. Tong et al. [23] developed a hidden Markov Model to identify the measurement disturbances and then adjust the noise covariance adaptively. Feng et al. [24] proposed a two-step correction scheme, in which the magnetometer output is used to correct the estimated direction of the magnetic field, but if the difference between the norm of the sensor output and the reference value is greater than a threshold, the correction will not be executed. In fact, the above methods apply a same strategy, i.e., detecting magnetic disturbances according to some feature, e.g., the norm, of the measured magnetic field, and reducing the measurement update weight in real time when disturbances happen. The drawback of this strategy is that the magnetometer measurement update cannot provide effective and timely correction for the yaw estimation if magnetic disturbances last for a long time. In addition, Sabatini [25] proposed an extended Kalman Filter, which compensated magnetic disturbances by including them in the state vector. The filter models magnetic disturbances by a first-order Gauss-Markov vector random process with independent components, and assume the motion acceleration is approximately zero. In fact, the actual magnetic disturbances can hardly be modelled effectively, and the assumption about the motion acceleration is not suitable for land vehicles.

Another problem in yaw estimation is its impact on attitude estimation. In some fusion algorithms, the magnetometer output is also used to correct the attitude estimation, and thus induce estimation errors caused by magnetic disturbances. To address this problem, some algorithms restrict the use of the magnetometer output to yaw estimation. In [9,10], orientation quaternion is obtained through multiplication of a series of decoupled quaternion factors, and the result can be used as the measurement for a Kalman filter with a two-layer structure [9]. Suh [26] proposed a two-step measurement update for an indirect Kalman filter, where the magnetometer measurement update only affected the yaw estimation. Afterwards, Suh et al. [27] proposed a new measurement equation for the indirect Kalman filter, which can greatly reduce the impact of the magnetometer measurement update on attitude estimation and is easier to implement than the two-step measurement update algorithm.

Obviously, both of the mentioned problems are due to magnetic disturbances. This paper aims to enhance the ability of the yaw estimation to deal with magnetic disturbances. One basic idea of this paper is that use not only the information from the sensors, but also the characters of vehicles motion to improve yaw estimation. We think motion characters can provide some independent information, which can be used as supplement to the sensors information. We note that the yaw of a vehicle running along a straight road remains basically unchanged, and this character can be utilized to improve the yaw estimation as a supplement to the magnetometer measurement update. To achieve this, it should be known whether a vehicle is running straight. A straightforward way to determine the vehicle status is comparing the gyroscope output against some preset parameters, but the gyroscope output suffers from various measurement errors and vehicle bumps. Considering the turning motion cause changes of MARG outputs, we can determine the vehicle status by analyzing the correlation between outputs from different sensors. In addition, we can reform the measurement update process of a direct Kalman filter using the condition proved in [26] and thus make the attitude estimation immune to magnetic disturbances. In fact, more accurate attitude estimation is also helpful to improve the yaw estimation because they are coupled in the time propagation. Motivated by above discussion, we propose an improved yaw estimation algorithm, and the main contributions of this paper are as follows:

(1) A new measurement update robust to magnetic disturbances is introduced, and its weight can be adjusted according to the vehicle status. The correlation coefficients between outputs from a MARG sensor are analyzed based on the vehicle kinematic model and Coriolis theorem, and a vehicle status marker is constructed.
(2) A new two-step measurement update method is designed. The method implements measurement updates in two successive steps, and make a special processing of the magnetometer measurement update to eliminate its impact on attitude estimation.

In this paper, we construct a yaw estimation algorithm using a typical linear Kalman filter to implement basic quaternion estimation and applying the conventional strategy of reducing the measurement weight to deal with magnetic disturbances. This algorithm is referred to as conventional algorithm. Then, the conventional algorithm is improved with the new measurement update and the new two-step measurement update method. Finally, the performances of the improved algorithm are evaluated through comparing its results against that of the conventional algorithm in experiments. The rest of this paper is organized as follows: Section 2 describes a conventional yaw estimation algorithm. Section 3 details the improved algorithm. Section 4 provides the experiment results and discussion. Finally, the work is concluded in Section 5.

## 2. Conventional Algorithm

### 2.1. Preliminaries

Because of not suffering from the gimbal lock, low dimension, and offering a linear formulation of the orientation dynamics [9], quaternion is widely used for orientation representation. Any orientation of a body frame with respect to a reference frame can be represented by a unit quaternion q defined as:

(1) $$q={\left[\begin{array}{cccc} q_{0} & q_{1} & q_{2} & q_{3} \end{array}\right]}^{T}={\left[\begin{array}{cccc} \cos\frac{\alpha }{2} & e_{x}\sin\frac{\alpha }{2} & e_{y}\sin\frac{\alpha }{2} & e_{z}\sin\frac{\alpha }{2} \end{array}\right]}^{T}\;$$

where $q_{0}$ is the scalar part; ${\left[\begin{array}{ccc} q_{1} & q_{2} & q_{3} \end{array}\right]}^{T}$ is the vector part; α is the rotation angle; ${\left[\begin{array}{ccc} e_{x} & e_{y} & e_{z} \end{array}\right]}^{T}$ is the unit vector that represents the rotation axis. In quaternion estimation algorithms, the gyroscope output is used to depict the quaternion dynamics; therefore, the gyroscope bias is an important factor that affects the estimation accuracy.

In this section, we describe a Kalman filter-based yaw estimation algorithm with the unit quaternion and gyroscope bias as states. This algorithm, referred to as conventional algorithm, provides the basement and benchmark for the improved algorithm presented in the next section. The conventional algorithm is based on a typical linear Kalman filter presented in [16]. In addition, adaptive measurement weights are applied to deal with measurement disturbances, and the yaw is computed with the quaternion estimation.

The detailed derivations of the system model are presented in [16]; for conciseness, we only list the results and give necessary explanations in this section. Note that we use a different quaternion definition from that in [16] (The scalar part is the first component of a quaternion, whereas in [16], the scalar part is the last component); therefore, we rewrite the concerned equations accordingly, which will not affect the performances of the algorithm.

In this paper, the reference frame is the East, North, Up frame; the body frame is the Right, Forward, Above frame; the sensor frame is assumed aligned with the body frame; the ZXY sequence of Euler angles is chosen, and the yaw, pitch, and roll are respectively *z*-axis rotation angle, *x*-axis rotation angle, and *y*-axis rotation angle. For a clear writing, we define some notations that will be used throughout this paper as follows.

Vectors: x is the state vector; b is the gyroscope bias; $m_{r}$ and $g_{r}$ are respectively the geomagnetic filed and the gravity resolved in the reference frame; m, ω, and a are respectively the output of magnetometer, gyroscope, and accelerometer.

Matrices: $C_{r}^{b}$ is the rotation matrix from the reference frame to the body frame; 0 and I are respectively null matrices and identity matrices, and their subscripts indicate dimensions, for example, the dimensions of $0_{3\times 4}$, $0_{3}$, and $I_{4}$ are respectively 3 × 4, 3 × 3, and 4 × 4; [v×] is the skew-symmetric matrix of vector v.

Subscripts: *x*, *y*, and *z* denote respectively the *x*-axis, *y*-axis, and *z*-axis component of a vector; *k* denotes the time step.

### 2.2. System Model

The state vector, composed of the unit quaternion and gyroscope bias, is defined as:

(2) $$x={\left[\begin{array}{cc} q^{T} & b^{T} \end{array}\right]}^{T}\;$$

Based on the well-known quaternion dynamics model and state augmentation, the process equation is written as:

(3a) $$x_{k+1}=\Psi_{k}x_{k}+\Gamma_{k}n_{k}\;$$

where:

(3b) $$\Psi_{k}=\left[\begin{array}{cc} \Phi \left(\theta_{k}\right) & -\frac{\Delta t}{2}\Xi_{k}\\ 0_{3\times 4} & I_{3} \end{array}\right]\;$$

(3c) $$\Phi \left(\theta_{k}\right)=\exp\left(\frac{1}{2}\left[\begin{array}{cc} 0 & -\theta_{k}^{T}\\ \theta_{k} & -\left[\theta_{k}\times \right] \end{array}\right]\right)\;$$

(3d) $$\theta_{k}=\omega_{k}\Delta t\;$$

(3e) $$\Xi_{k}=\left[\begin{array}{c} -q_{v,k}^{T}\\ \left[q_{v,k}\times \right]+q_{0,k}I_{3} \end{array}\right]\;$$

(3f) $$\Gamma_{k}=\left[\begin{array}{ccc} -\frac{1}{2}\Xi_{k} & -\frac{1}{2}\Xi_{k} & 0_{4\times 3}\\ 0_{3} & 0_{3} & I_{3} \end{array}\right]\;$$

In (3), $\Psi_{k}$ is the transition matrix; $\Gamma_{k}$ is the process noise input matrix; Δt is the sample interval; $q_{v}$ is the vector part of the quaternion state, and $n_{k}$ is the gyroscope noise with covariance matrix $\mathrm{diag}\left(\sigma_{\omega }^{2},\sigma_{\omega }^{2},\sigma_{\omega }^{2}\right)$.

The linear pseudo-measurement equation of the accelerometer is:

(4a) $$0_{4\times 1}=H_{a,k+1}^{}x_{k+1}-\frac{1}{2}\Xi_{k+1}\delta a_{k+1}\;$$

where:

(4b) $$H_{a,k+1}^{}=\left[\begin{array}{cc} \left[\begin{array}{cc} 0 & -d_{a,k+1}^{T}\\ d_{a,k+1} & -\left[s_{a,k+1}\times \right] \end{array}\right] & 0_{4\times 3} \end{array}\right]\;$$

(4c) $$s_{a,k+1}=\frac{1}{2}(a_{k+1}+g_{r})\;$$

(4d) $$d_{a,k+1}=\frac{1}{2}(a_{k+1}-g_{r})\;$$

(4e) $$\delta a_{k+1}=a_{k+1}^{}-C_{r}^{b}g_{r}\;$$

In (4), $H_{a,k+1}^{}$ is the measurement matrix, and $-\frac{1}{2}\Xi_{k+1}\delta a_{k+1}$ is the measurement noise. The covariance matrix of $\delta a_{k+1}$ is $\sigma_{a}^{2}I_{3}$.

Similarly, the linear pseudo-measurement equation of the magnetometer is:

(5a) $$0_{4\times 1}=H_{m,k+1}^{}x_{k+1}-\frac{1}{2}\Xi_{k+1}\delta m_{k+1}\;$$

where:

(5b) $$H_{m,k+1}^{}=\left[\begin{array}{cc} \left[\begin{array}{cc} 0 & -d_{m,k+1}^{T}\\ d_{m,k+1} & -\left[s_{m,k+1}\times \right] \end{array}\right] & 0_{4\times 3} \end{array}\right]\;$$

(5c) $$s_{m,k+1}=\frac{1}{2}(m_{k+1}+m_{r})\;$$

(5d) $$d_{m,k+1}=\frac{1}{2}(m_{k+1}-m_{r})\;$$

(5e) $$\delta m_{k+1}=m_{k+1}^{}-C_{r}^{b}m_{r}\;$$

In (5), $H_{m,k+1}^{}$ is the measurement matrix, and $-\frac{1}{2}\Xi_{k+1}\delta m_{k+1}$ is the measurement noise. The covariance matrix of $\delta m_{k+1}$ is $\sigma_{m}^{2}I_{3}$.

From (4) and (5), the overall measurement equation can be written as:

(6a) $$0_{8\times 1}=H_{k+1}^{}x_{k+1}-\frac{1}{2}\mathrm{diag}\left(\Xi_{k+1},\Xi_{k+1}\right){\left[\begin{array}{cc} \delta a_{k+1}^{T} & \delta m_{k+1}^{T} \end{array}\right]}^{T}\;$$

where:

(6b) $$H_{k+1}^{}=\left[\begin{array}{c} H_{a,k+1}^{}\\ H_{m,k+1}^{} \end{array}\right]\;$$

The noise covariance matrices of the process and measurement equations are written as:

(7) $$\mathrm{cov}\left(\Gamma_{k}n_{k}\right)=Q_{k}\;$$

(8) $$\mathrm{cov}\left(-\frac{1}{2}\Xi_{k+1}\delta a_{k+1}\right)=\rho_{a,k+1}^{}R_{a,k+1}^{}\;$$

(9) $$\mathrm{cov}\left(-\frac{1}{2}\Xi_{k+1}\delta m_{k+1}\right)=\rho_{m,k+1}^{}R_{m,k+1}^{}\;$$

where $\rho_{a}^{}$ and $\rho_{m}^{}$ are adaptive weight coefficients, which can be adjusted in real time. The computation formulas for $Q_{k}$, $R_{a,k+1}^{}$, and $R_{m,k+1}^{}$ can be found in [16]. From (8) and (9), the noise covariance matrix of (6a) can be written as:

(10) $$R_{k+1}=\mathrm{diag}\left(\rho_{a,k+1}^{}R_{a,k+1}^{},\rho_{m,k+1}^{}R_{m,k+1}^{}\right)\;$$

### 2.3. Adaptive Fusion Algorithm Based on Kalman Filter

Filter Initialization: Set initial values for state vector estimation ${\hat{x}}_{}$, i.e., $\hat{q}$ and $\hat{b}$, and error covariance matrix $P_{}$.

Time Propagation: The process model, gyroscope output and posteriori estimation (or the initial estimation) at step *k* is used to compute the priori estimation at step *k* + 1 by:

(11a) $${\hat{x}}_{k+1/k}=\Psi_{k}{\hat{x}}_{k}\;$$

(11b) $$P_{k+1/k}=\Psi_{k}P_{k}\Psi_{k}^{T}+Q_{k}\;$$

Measurement weight adjustment: To deal with motion accelerations and magnetic disturbances, the weight coefficients $\rho_{a}$ and $\rho_{m}$ are adjust in real time according to the disturbance intensity, which is equivalent to adjust the measurement weight. The adjusting expressions are:

(12) $$\rho_{a,k+1}=\exp(\lambda_{a}\left|\left\|a_{k+1}\right\|-\left\|g_{r}\right\|\right|/\left\|g_{r}\right\|)\;$$

(13) $$\rho_{m,k+1}=\exp(\lambda_{m}\left|\left\|m_{k+1}\right\|-\left\|m_{r}\right\|\right|/\left\|m_{r}\right\|)\;$$

where the relative distances between the norms of measured vectors ($a_{k+1}$, $m_{k+1}$) and reference vectors ($g_{r}$, $m_{r}$) are used to represent the disturbance intensity, and the exponential functions are used to map the disturbance intensity to weight coefficients. The function of the parameters $\lambda_{a}$ and $\lambda_{m}$ is to adjust the mapping relations. If the weight coefficient should be more sensitive to the disturbance intensity, the corresponding parameter should be increased, otherwise, it should be reduced. The values used for $\lambda_{a}$ and $\lambda_{m}$ can be determined experimentally. The exponential function instead of a linear function is applied because the former can reduce the measurement weight more quickly when the relative distance increases [28].

Measurement Update: The measurement model, magnetometer and accelerometer outputs and the priori estimation at step *k* + 1 is used to compute the posteriori estimation at step *k* + 1 by:

(14a) $$K_{k+1}=P_{k+1/k}H_{k+1}^{T}{\left(H_{k+1}^{}P_{k+1/k}H_{k+1}^{T}+R_{k+1}\right)}^{-1}\;$$

(14b) $${\hat{x}}_{k+1}={\hat{x}}_{k+1/k}-K_{k+1}H_{k+1}{\hat{x}}_{k+1/k}\;$$

(14c) $$P_{k+1}=(I_{7}-K_{k+1}H_{k+1})P_{k+1/k}\;$$

Unit Constraint: To preserve the unit-norm property of the quaternion estimation, the updated quaternion is normalized by:

(15) $${\bar{q}}_{k+1}={\hat{q}}_{k+1}/\left\|{\hat{q}}_{k+1}\right\|\;$$

### 2.4. Yaw Computation

The rotation matrix from the reference frame to the body frame can be represented as a function of either Euler angles or a unit quaternion, and the expressions are respectively:

(16) $$C_{r}^{b}(\psi ,\phi ,\gamma )=\left[\begin{array}{ccc} c\gamma c\psi +s\gamma s\psi s\phi & -c\gamma s\psi +s\gamma c\psi s\phi & -s\gamma c\phi \\ s\psi c\phi & c\phi c\psi & s\phi \\ s\gamma c\psi -c\gamma s\psi s\phi & -s\gamma s\psi -c\gamma c\psi s\phi & c\gamma c\phi \end{array}\right]\;$$

(17) $$C_{r}^{b}(q)=\left[\begin{array}{ccc} q_{0}^{2}+q_{1}^{2}-q_{2}^{2}-q_{3}^{2} & 2(q_{1}q_{3}+q_{0}q_{3}) & 2(q_{1}q_{3}-q_{0}q_{2})\\ 2(q_{1}q_{2}-q_{0}q_{3}) & q_{0}^{2}-q_{1}^{2}+q_{2}^{2}-q_{3}^{2} & 2(q_{2}q_{3}+q_{0}q_{1})\\ 2(q_{1}q_{3}+q_{0}q_{2}) & 2(q_{2}q_{3}-q_{0}q_{1}) & q_{0}^{2}-q_{1}^{2}-q_{2}^{2}+q_{3}^{2} \end{array}\right]\;$$

where ψ, ϕ, and γ are respectively the yaw, pitch, and roll; s and c denote sine and cosine function, respectively.

Using the first two elements of the second row of C(q), denoted as $C_{21}$ and $C_{22}$ respectively, and defining the range of the yaw as (−180°, 180°], we obtain the following formulas for yaw computation:

(18) $$\hat{\psi }=\{\begin{array}{ll} \arctan(C_{21}/C_{22}) & C_{22}>0\\ \arctan(C_{21}/C_{22})+180^{\circ } & C_{22}<0,C_{21}>0\\ \arctan(C_{21}/C_{22})-180^{\circ } & C_{22}<0,C_{21}<0 \end{array}\;$$

## 3. Improved Algorithm

In this section, we keep the time propagation step of the conventional algorithm unchanged, and improve its measurement update step in two ways. Firstly, to improve the accuracy of the yaw estimation in the presence of magnetic disturbances, a measurement equation of the gyroscope bias is derived and its weight can be adjusted according to the vehicle status. Secondly, we design a two-step measurement update method to eliminate the impact of the magnetometer measurement update on the attitude estimation.

### 3.1. New Measurement Equation and Its Adaptive Weight

The gyroscope output can be written as:

(19a) $$\omega =\omega_{b}+b+n_{1}\;$$

and its *z*-axis component is:

(19b) $$\omega_{z}=\omega_{bz}+b_{z}+n_{1z}\;$$

where $\omega_{b}$ is the angular rate resolved in the body frame, and $n_{1}$ is a white Gaussian noise vector. When vehicles are running straight, $\omega_{bz}$ can be regarded as zero and (19b) can be rewritten as:

(20a) $$b_{z}=H_{\omega }x_{}+n_{1z}\;$$

where:

(20b) $$H_{\omega }=\left[\begin{array}{cc} 0_{1\times 6} & 1 \end{array}\right]\;$$

Equation (20a) is a new measurement equation named as gyroscope measurement, and its characters are as follows. Firstly, it only updates the gyroscope bias estimation in measurement update, but it will affect the quaternion estimation in time propagation through (11); specifically speaking, it will constrain the yaw estimation from changing by correcting the *z*-axis angular rate to zero. Secondly, it is immune to magnetic disturbances because the latter cannot impact the gyroscope output. Thirdly, the gyroscope measurement only hold true in running straight status, in other words, its validity depends on the vehicle status.

Note that, the yaw is computed using quaternion estimation, and all the gyroscope bias components can impact the quaternion propagation. But, in the fusion algorithm, the quaternion estimation is not only based on propagation, but also on measurement update. The yaw derivative [12] can be written as:

(21) $$\dot{\psi }=\frac{\sin\gamma }{\cos\phi }\omega_{bx}-\frac{\cos\gamma }{\cos\phi }\omega_{bz}\;$$

Obviously, the yaw derivative is related to pitch, roll, $\omega_{bx}$, and $\omega_{bz}$. In the fusion algorithm, the estimation of pitch and roll will be corrected by the accelerometer measurement (in the form of correcting quaternion), which is immune to magnetic disturbances. The two-step measurement update method which will be introduced in Section 3.2 makes pitch and roll estimation immune to the errors induced by magnetometer measurement update. In addition, generally speaking, the pitch and roll are small for land vehicles, hence the absolute value of the coefficient of $\omega_{bz}$ is greater than that of $\omega_{bx}$. Therefore, the z-axis bias component is of more importance, especially in magnetically disturbed environments. Equation (21) is based on Euler angles, but it should be noted that both quaternion and Euler angles are representation of orientation, and orientation obeys a unique dynamic rule. In other words, the quaternion propagation models is different from that of Euler angles, but when they are used to represent a same orientation parameter, for example yaw, the parameter obeys a same dynamic rule no matter what representation is used.

The above characteristics suggest that the gyroscope measurement can be used to correct the yaw estimation in the presence of magnetic disturbances when vehicle is running straight. In practice, a vehicle cannot always run on a straight road, and even on a straight road the yaw may fluctuate. In order to employ this measurement correctly, we define the covariance of the measurement noise $n_{1z}$ as $\rho_{\omega }^{}\sigma_{\omega }^{2}$, where $\rho_{\omega }^{}$, similar to $\rho_{a}^{}$ and $\rho_{m}^{}$, works as a adaptive weight coefficient that can be adjusted in real time. Obviously, the adjusting principle should be that the higher the extent of running straight is, the smaller the $\rho_{\omega }^{}$ is.

Now, the problem is how to determine the extent of running straight. To address this problem, we analyze the correlations between different sensor outputs. The accelerometer output can be written as:

(22) $$a=a_{m}-C_{r}^{b}g_{r}+n_{2}\;$$

where $a_{m}$ is the motion acceleration, and $n_{2}$ is a noise vector. According to the vehicle kinematic model [29], $a_{m}$ is given by:

(23) $$a_{m}={\dot{v}}_{b}+\omega_{b}\times v_{b}\;$$

where $v_{b}$ is the velocity of the vehicle in the body frame.

**Figure 1:**
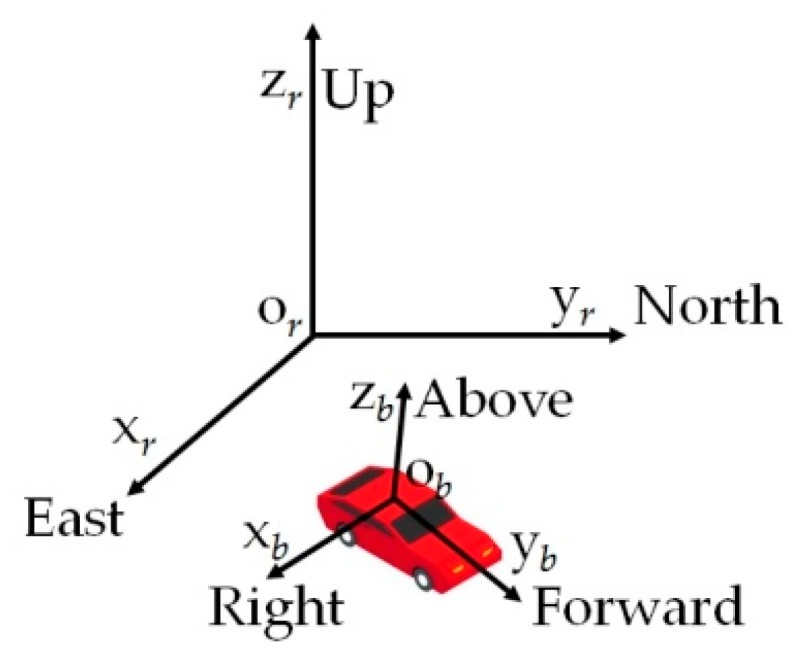
Reference frame $o_{r}x_{r}y_{r}z_{r}$ and body frame $o_{b}x_{b}y_{b}z_{b}$.

The reference fame and body frame are shown in Figure 1. For a land vehicle, the running direction can be arbitrary in reference frame, but in the body frame, which is attached to the vehicle, its running direction is always forward, although the forward direction may change in reference frame. The “forward direction” is the *y* direction in the body frame defined in this paper. Dissanayake et al. [30] have pointed out that, when the vehicle does not jump off the ground and does not slide on the ground, velocity of the vehicle in the plane perpendicular to the forward direction is zero, hence $v_{bx}$ and $v_{bz}$ can be assumed as zero. Using this assumption and substituting (16) and (23) into (22), we simplify the *x*-axis component of (22) as:

(24) $$a_{x}=-v_{by}\omega_{bz}-gs\gamma c\phi +n_{2x}\;$$

In practical situation, the assumption is somewhat violated due to the presence of side slip during cornering and vibrations caused by the engine and suspension system [30]. When the assumption is violated, the side slips and vibrations will cause corresponding accelerations, but these accelerations can be regarded as noise and a part of $n_{2x}$ in (24).

The magnetic field resolved in the body frame, denoted as $m_{b}$, can be written as:

(25) $$m_{b}=C_{r}^{b}(m_{r}+d_{r})\;$$

where $d_{r}$ is the magnetic disturbances resolved in the reference frame. According to the Coriolis theorem, the change rate of $m_{b}$ in body frame can be written as:

(26) $${\dot{m}}_{b}=C_{r}^{b}({\dot{m}}_{r}+{\dot{d}}_{r})-\omega_{b}\times m_{b}\;$$

Because ${\dot{m}}_{r}=0$, the *x*-axis and *y*-axis components of ${\dot{m}}_{b}$ can be written as:

(27a) $${\dot{m}}_{bx}=C_{r1\cdot }^{b}\cdot {\dot{d}}_{r}+\omega_{bz}m_{by}-\omega_{by}m_{bz}\;$$

(27b) $${\dot{m}}_{by}=C_{r2\cdot }^{b}\cdot {\dot{d}}_{r}-\omega_{bz}m_{bx}+\omega_{bx}m_{bz}\;$$

where $C_{r1\cdot }^{b}$ and $C_{r2\cdot }^{b}$ denote the first row and second row of $C_{r}^{b}$ respectively. We rewrite (27) as:

(28a) $${\dot{m}}_{bx}/m_{by}=\omega_{bz}+C_{r1\cdot }^{b}\cdot {\dot{d}}_{r}/m_{by}-\omega_{by}m_{bz}/m_{by}\;$$

(28b) $$-{\dot{m}}_{by}/m_{bx}=\omega_{bz}-C_{r2\cdot }^{b}\cdot {\dot{d}}_{r}/m_{bx}+\omega_{bx}m_{bz}/m_{bx}\;$$

When the angle between the road plane and the horizontal plane remains constant and the vehicle do not vibrate, $\omega_{by}$ and $\omega_{bx}$ are zero. In practice, the angle between the two planes may change, however, in general, the rate of change will be small, and the duration of the vehicle attitude change is also very short. Hence, the corresponding $\omega_{by}$ and $\omega_{bx}$ will be small and close to zero for most of the time. In the case of vibration, which can be caused by bumps or ditches, $\omega_{by}$ and $\omega_{bx}$ may have large absolute value, but they will oscillate with high frequency. Hence, regarding the last item of the right side as noise and using the magnetometer output and difference to approximate the left side of (28), we obtain:

(29a) $$\left(m_{x,k}-m_{x,k-1}\right)/\left(m_{y,k}\Delta t\right)=\omega_{bz}+C_{r1\cdot }^{b}\cdot {\dot{d}}_{r}/m_{by}+n_{3}\;$$

(29b) $$-(m_{y,k}-m_{y,k-1})/\left(m_{x,k}\Delta t\right)=\omega_{bz}-C_{r2\cdot }^{b}\cdot {\dot{d}}_{r}/m_{bx}+n_{4}\;$$

where $n_{3}$ and $n_{4}$ are noises. Because $m_{y,k}$ and $m_{x,k}$ may be equal or close to zero and thus cause numerical instability, we define Δm as:

(30) $$\Delta m_{k}=\{\begin{array}{r} \left(m_{x,k}-m_{x,k-1}\right)/\left(m_{y,k}\Delta t\right)\begin{array}{cc} & \left|m_{y,k}\right|>\left|m_{x,k}\right| \end{array}\\ -(m_{y,k}-m_{y,k-1})/\left(m_{x,k}\Delta t\right)\begin{array}{cc} & \left|m_{y,k}\right|\leq \left|m_{x,k}\right| \end{array} \end{array}\;$$

Equations (19b) and (24) show that the correlation between $\omega_{z}$ and $a_{x}$ will be low if $\omega_{bz}$ is stable; otherwise, the correlation will be high. Equations (19b), (29), and (30) show that $\omega_{z}$ and Δm have similar relationships; more importantly, $d_{r}$ will not affect the correlation between $\omega_{z}$ and Δm if it is constant and will not affect the correlation markedly except for an abrupt changing. Hence, the correlation between $\omega_{z}$ and Δm is robust to magnetic disturbances.

Considering $\omega_{bz}$ is stable in running straight status and unstable in turning status, we construct a vehicle status marker *c*, whose expression is:

(31) $$c=\max\left(0,\frac{1}{2}\mathrm{cor}\left(\omega_{z,N},\Delta m_{N}\right)-\frac{1}{2}\mathrm{cor}\left(\omega_{z,N},a_{x,N}\right)\right)\;$$

where cor(⋅) estimates the correlation coefficient using the samples of the sensor outputs, and subscript *N* denotes the *N* points samples from k−N+1 to k. In fact, in running straight status, MARG sensor outputs are stable except for uncorrelated noises; whereas in turning status, the outputs change, and the output from different sensor is correlative because their changes are caused by the same reason i.e., the vehicle is turning. The rationale of (31) is that use estimated correlation coefficients to distinguish status. In (31), the weighted sum combine the two correlation coefficient to make full use of the correlation between different sensors, and the second term of the sum is minus because $\omega_{z}$ is negatively correlated to $a_{x}$ when $\omega_{bz}$ is changing. Considering the sum is theoretically nonnegative, we set c to zero when correlation coefficient estimation errors result in a negative sum. Similar to $\rho_{a}$ and $\rho_{m}$, the adjusting expression for $\rho_{\omega }$ is:

(32) $$\rho_{\omega ,k+1}=\exp(\lambda_{\omega }c)\;$$

where $\lambda_{\omega }$ is a coefficient that transforms the status marker to a proper range.

### 3.2. Two-Step Measurement Update Method

This part presents the two-step measurement update method, and the concerned parameters of the first and second update step are respectively denoted by subscript f and s in the following.

First Step: The accelerometer and gyroscope measurement updates are implemented in this step. The measurement matrix and noise covariance matrix are respectively:

(33) $$H_{f,k+1}^{}=\left[\begin{array}{c} H_{a,k+1}^{}\\ H_{\omega }^{} \end{array}\right]\;$$

(34) $$R_{f,k+1}=\mathrm{diag}\left(\rho_{a,k+1}^{}R_{a,k+1}^{},\rho_{\omega ,k+1}^{}\sigma_{\omega }^{2}\right)\;$$

The update expressions are:

(35a) $$K_{f,k+1}=P_{k+1/k}H_{f,k+1}^{T}{\left(H_{f,k+1}^{}P_{k+1/k}H_{f,k+1}^{T}+R_{f,k+1}^{}\right)}^{-1}\;$$

(35b) $${\hat{x}}_{f,k+1}={\hat{x}}_{k+1/k}+K_{f,k+1}(Z_{f,k+1}^{}-H_{f,k+1}{\hat{x}}_{k+1/k})\;$$

(35c) $$P_{f,k+1}=(I_{7}-K_{f,k+1}H_{f,k+1})P_{k+1/k}\;$$

where:

(35d) $$Z_{f,k+1}^{}=\left[\begin{array}{c} 0_{4\times 1}\\ \omega_{z,k+1} \end{array}\right]\;$$

Second Step: In this step, the magnetometer measurement update is implemented and the ultimate quaternion estimation is computed. The measurement matrix and noise covariance matrix are respectively:

(36) $$H_{s,k+1}^{}=H_{m,k+1}^{}\;$$

(37) $$R_{s,k+1}=\rho_{m,k+1}R_{m,k+1}\;$$

The update expressions are:

(38a) $$K_{s,k+1}=P_{f,k+1}H_{s,k+1}^{T}{\left(H_{s,k+1}^{}P_{f,k+1}H_{s,k+1}^{T}+R_{s,k+1}^{}\right)}^{-1}\;$$

(38b) $${\hat{x}}_{s,k+1}={\hat{x}}_{f,k+1}-K_{s,k+1}H_{s,k+1}{\hat{x}}_{f,k+1}\;$$

(38c) $$P_{s,k+1}=(I_{7}-K_{s,k+1}H_{s,k+1})P_{f,k+1}\;$$

Extracting quaternion part of ${\hat{x}}_{f,k+1}$ and ${\hat{x}}_{s,k+1}$, we obtain ${\hat{q}}_{f,k+1}$ and ${\hat{q}}_{s,k+1}$. Define $q_{\sigma }$ as:

(39) $$q_{\sigma }={\hat{q}}_{f,k+1}^{*}⊗{\hat{q}}_{s,k+1}\;$$

where ${\hat{q}}_{f,k+1}^{*}$ is the conjugate quaternion of ${\hat{q}}_{f,k+1}$ and the symbol ⊗ is the quaternion multiplication operator. The conjugate quaternion of a unit quaternion represents the inverse rotation and a sequence of rotations can be represented by quaternion multiplication; therefore, $q_{\sigma }$ can be viewed as the correction quaternion induced by the magnetometer measurement update. In theory, $q_{\sigma }$ should only correct the yaw estimation; however, it also modify the attitude estimation in practice. The corresponding rotation matrix equation to Equation (39) can be written as:

(40) $$C({\hat{q}}_{s,k+1})=C(q_{\sigma })C({\hat{q}}_{f,k+1})\;$$

The condition for $C({\hat{q}}_{s,k+1})$ and $C({\hat{q}}_{f,k+1})$ to share a same attitude [26] is:

(41) $$q_{\sigma v}\times C{\left({\hat{q}}_{f,k+1}\right)}_{\cdot 3}^{}=0_{3\times 1}\;$$

where $q_{\sigma v}^{}$ is the vector part of $q_{\sigma }^{}$ and $C{\left({\hat{q}}_{f,k+1}\right)}_{\cdot 3}^{}$ is the third column of $C({\hat{q}}_{f,k+1})$. The geometric meaning of (41) is that the rotation axis of $q_{\sigma }$ should be parallel to the z-axis of the reference frame. Based on this condition and (1), a new correction quaternion ${\hat{q}}_{\sigma }$ is defined as:

(42a) $${\hat{q}}_{\sigma }={\left[\begin{array}{cc} \cos(\alpha ) & \sin(\alpha )C{\left({\hat{q}}_{f,k+1}\right)}_{\cdot 3}^{T} \end{array}\right]}^{T}\;$$

where:

(42b) $$\alpha =\arccos(q_{\sigma ,0})e\cdot C{\left({\hat{q}}_{f,k+1}\right)}_{\cdot 3}^{}\;$$

(42c) $$e=q_{\sigma v}^{}/\sin(\arccos(q_{\sigma ,0}))\;$$

Note that ${\hat{q}}_{\sigma }$ is actually the projection of $q_{\sigma }$ on the direction depicted by $C{\left({\hat{q}}_{f,k+1}\right)}_{\cdot 3}^{}$, and this treatment makes ${\hat{q}}_{\sigma }$ satisfy (41). Finally, the ultimate quaternion estimation is computed by:

(43) $${\hat{q}}_{k+1}={\hat{q}}_{f,k+1}⊗{\hat{q}}_{\sigma }\;$$

### 3.3. Complete Improved Yaw Estimation Algorithm

**Figure 2:**
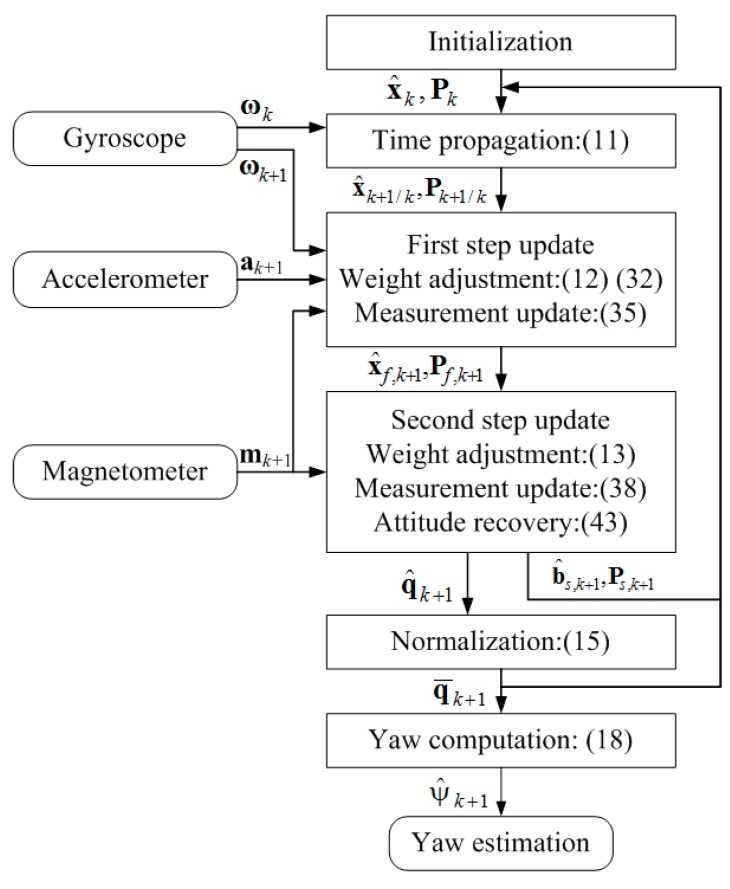
Improved yaw estimation algorithm.

Adding the adaptive gyroscope measurement update to the conventional algorithm and adopting the two-step measurement update method, we obtain the improved algorithm shown in Figure 2. Note that the improved algorithm preserves the linearity and all its measurement updates have an adaptive weight.

## 4. Experiments

In this section, we evaluate the performances of the improved algorithm experimentally. The real data from a MARG sensor mounted on a test vehicle are processed by the conventional and improved algorithm, respectively. Then, we analyze the performances of the vehicle status marker, gyroscope measurement update, two-step measurement update method, and yaw estimation by comparing the results of the improved algorithm against that of the conventional algorithm and reference values.

### 4.1. Experimental Setup and Parameters

**Figure 3:**
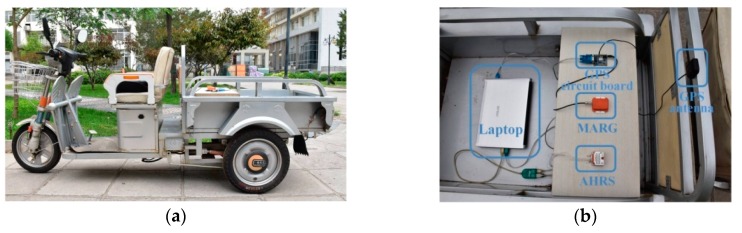
Experimental setup. (**a**) Test vehicle. (**b**) Experimental devices.

The test vehicle and experimental devices are shown in Figure 3. A Motion Tracker MTi-28A53G35 (Xsens, Enschede, The Netherlands) [31] was used as the MARG sensor. The Global Position System (GPS) unit (Unicoremm, Beijing, China) provided reference values of longitude, latitude, and forward velocity. 3-axis gyro module STIM210 (Sensonor AS, Horten, Norway) was used as the attitude and heading reference system (AHRS), which provided reference values of the yaw and attitude. The initial value of the AHRS was computed with the static accelerometer and magnetometer outputs [32]. The laptop (Lenovo, Beijing, China) supplied power for the MARG sensor, GPS unit, and the AHRS, and logged data from them at 100Hz. The GPS unit, MARG sensor, and AHRS were mounted on the test vehicle with the sensor and AHRS frames aligned with the vehicle body frame. In experiments, the vehicle was driven along test trajectories, which consisted of straight lines, corners with different angles and a circular line, involving a full range of yaw. Note that the magnetic sensor had been calibrated inline according to the sensor manual [20]; hence, we assumed that the impact of the hard and soft iron effects had been eliminated.

We analyzed the MARG sensor output in static condition, and set the noise parameters as follows: $\sigma_{m}=0.0015$, $\sigma_{\omega }=0.0056$ rad/s, $\sigma_{a}=0.008$ m/s^2^. We found the proper values for $\lambda_{m}$, $\lambda_{\omega }$, and $\lambda_{a}$ by trial and error, and set them to 20, 15, and 50 respectively. The estimation of $q_{0}$ was computed with the initial outputs of the magnetometer and accelerometer; the estimation of $b_{0}$ was set to the gyroscope output before the vehicle was started, and $P_{0}=100I_{7}$. The initial values of Q, $R_{a}^{}$, and $R_{m}^{}$ were computed with $q_{0}$ and $P_{0}$ [16].

### 4.2. Experimental Results and Discussion

#### 4.2.1. Vehicle Status Marker

**Figure 4:**
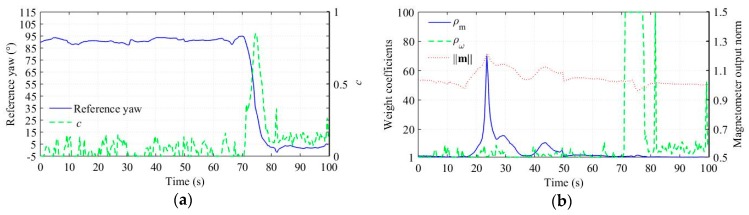
Adaptive weight coefficient based on the vehicle status. (**a**) Reference yaw and *c*. Because the reference yaw and c have different value range, two vertical axes are used for clarity with the right one corresponding to *c*. (**b**) $\rho_{m}$ and $\rho_{\omega }$. The magnetometer output norm is also plotted to show the relationship between magnetic disturbances and $\rho_{m}$, and, similar to (**a**), two vertical axes are used with the right one corresponding to ‖m‖. To obtain a proper vertical axe limit and thus the trends of curves can be showed clearly, the maximum value of the weight coefficients is set to 100.

To analyze the experimental results more clearly, we intercepted a piece of the MARG sensor outputs corresponding to a segment of the test trajectories. Using these data, we computed the status marker *c* and the adaptive weight coefficient $\rho_{\omega }$, which are shown in Figure 4. In Figure 4a, the reference yaw indicates that the vehicle undergone a turning (about 90°) between 70 s and 80 s, and mainly run straight with small fluctuation of yaw in other times. Corresponding to the reference yaw, *c* increases markedly between 70 s and 80 s; and oscillates with small values in other times. One key to computing c effectively is to select a proper *N*, because too many sample points will cause severely lagged weight adjustment, whereas too few sample points will reduce the computation accuracy. In the experiments, we set N=1/Δt.

#### 4.2.2. Gyroscope Measurement Update

**Figure 5:**
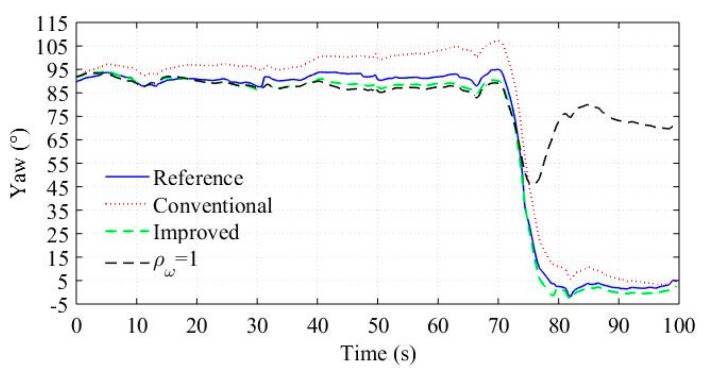
Yaw estimation.

We processed the sensor outputs using the conventional and improved algorithm, respectively, and the yaw estimation results are shown in Figure 5. The results demonstrate that the improved algorithm has better estimation accuracy, and the reasons may be analyzed as follows. In the conventional algorithm, the magnetometer measurement update corrects the yaw estimation from drifting, but the measurement weight will be adjusted down in the presence of magnetic disturbances (Figure 4b shows obvious differences between the norm of magnetometer output and one from about 15 s to 50 s implying the presence of magnetic disturbances). As a result, the correction effect attenuates and thus the yaw estimation drifts. In contrast, the gyroscope measurement update provides another correction in the improved algorithm, and more importantly, the correction can hardly be attenuated by magnetic disturbances as analyzed in Section 3. The adaptive weight coefficients shown in Figure 4b verify the function of the gyroscope measurement update: $\rho_{\omega }$ is much lower than $\rho_{m}$ in the presence of magnetic disturbances. In fact, $\rho_{\omega }$ represent the validity of the gyroscope measurement, which is not affected by magnetic disturbances. Hence, the gyroscope measurement can be used in magnetically disturbed environments. The real-time adjusting $\rho_{\omega }$ is critical for the gyroscope measurement to work effectively. Figure 5 also shows the yaw estimation result when $\rho_{\omega }$ is set to constant 1. The result demonstrates that the constant $\rho_{\omega }$ causes erroneous correction from the gyroscope measurement when the vehicle is turning, and hence results in significant estimation errors.

**Figure 6:**
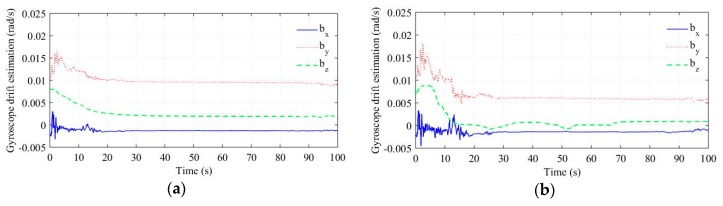
Gyroscope bias estimation. (**a**) Conventional algorithm. (**b**) Improved algorithm.

The gyroscope bias estimation results are shown in Figure 6. We estimated the reference value of $b_{z}$ by calculating the mean value of the gyroscope output in a not-moving interval, and the result was 0.00058 rad/s. Obviously, the $b_{z}$ estimation is updated more effectively in the improved algorithm, which is verified by its better yaw estimation accuracy.

In Section 3.1, we draw a conclusion based on Equation (21) that $b_{z}$ estimation is more important than $b_{x}$ and $b_{y}$ estimation in the fusion algorithm. To verify this conclusion based on experimental data, we constructed a conventional adaptive fusion algorithm using quaternion as state variables, and using Equation (6) as measurement equation. Note that, in this algorithm, the gyroscope bias is not estimated and compensated, and hence it will always impact the quaternion propagation. To examine the impact of the gyroscope bias, we added a constant bias on the reference angular rate provided by the AHRS and used the biased value to realize the quaternion propagation in the fusion algorithm.

We used this algorithm to estimate yaw for three times, and the constant bias were respectively set to [b, 0, 0], [0, b, 0], and [0, 0, b]. The results were denoted as **bx**, **by**, and **bz** respectively. Note that, in this process, all the parameters of the algorithm are same except for the constant bias. The root mean square (RMS) errors of the yaw estimation corresponding to different b are listed in Table 1. Obviously, *b_z_* has a more significant impact on yaw estimation.

It should be noted that the gyroscope measurement updates only provide a “partial” correction, in other words, they cannot provide absolute yaw information but that the yaw is unchanged to some extent, and the “extent” is indicated by the adaptive weight. Therefore, it is reasonable to employ the gyroscope measurement updates in combination with the magnetometer measurement updates. In addition, the gyroscope measurement updates only work effectively in running straight status. In practice, turning status is inevitable, but the duration of turning is relatively short, and running along a straight road is a more usual status for most land vehicles.

**Figure 7:**
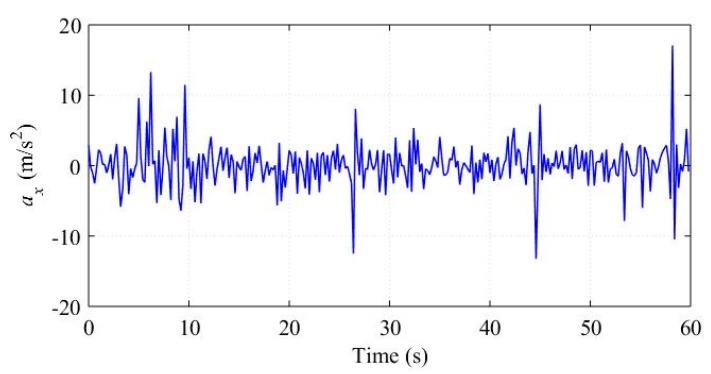
$a_{x}$ in bumps and ditches experiment.

**Figure 8:**
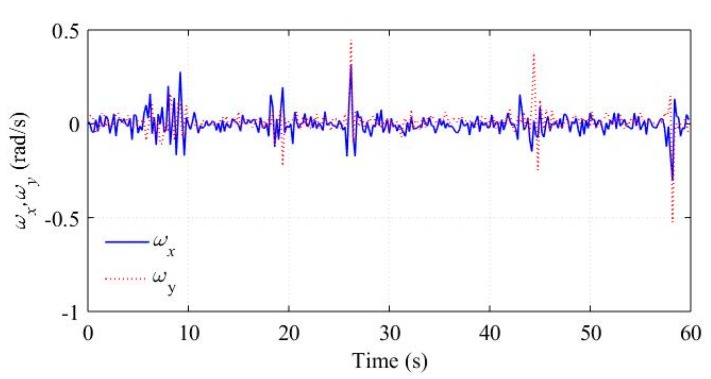
$\omega_{x}$ and $\omega_{y}$ in bumps and ditches experiment.

**Figure 9:**
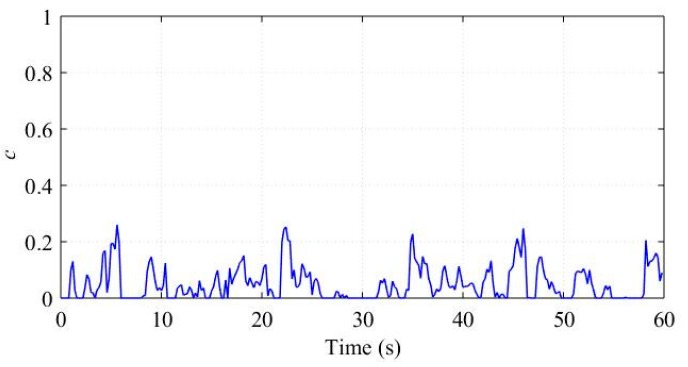
*c* in bumps and ditches experiment.

In practice, a running vehicle cannot avoid bumps and ditches, which will cause oscillations of $a_{x}$, $\omega_{x}$, and $\omega_{y}$. We assume these oscillations as part of the noise items in Equations (24) and (29) respectively, and construct the vehicle status marker *c* based on the correlations between different sensor outputs. To evaluate these noise assumptions, we analyzed a piece of the sensor outputs corresponding to a straight road with some bumps and ditches. The raw signals of $a_{x}$, $\omega_{x}$, and $\omega_{y}$ are shown in Figure 7 and Figure 8, respectively. The term *c* computed by Equation (31) is shown in Figure 9.

The vehicle met a bump or ditch at about 7 s, 19 s, 27 s, 45 s, and 58 s. Obviously, the bumps and ditches cause oscillations of the sensor outputs. Figure 9 demonstrate that the bumps and ditches do not affect *c* significantly, which displays small values and is consistent with the running straight status.

#### 4.2.3. Two-Step Measurement Update Method

**Figure 10:**
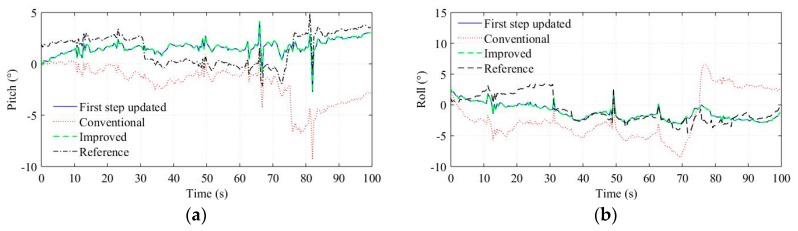
Attitude estimation. (**a**) Pitch estimation. (**b**) Roll estimation.

In the experiments, the test road is basically level except for some speed breaks. To evaluate the performances of the two-step measurement update method, we computed attitudes using the quaternion [33] from the first step update, the conventional algorithm and the improved algorithm respectively, and the results are shown in Figure 10. Obviously, the improved algorithm and the first step update have the same attitude estimation, which verify that the second step update do not modify the attitude estimation. In addition, the attitude estimation of the improved algorithm is more consistent with the reference values than the conventional algorithm. The comparison demonstrates that the two-step measurement update method eliminates the attitude estimation errors induced by the magnetometer measurement update. Besides magnetic disturbances, the magnetometer measurement error can also cause attitude estimation errors. For example, the attitude estimation of the conventional algorithm change incorrectly during the turning of the vehicle (70–80 s), and similar phenomena can be found in other turning processes, which, we think, is due to the dynamic errors of the magnetometer.

It should be noted that modifications of the attitude estimation made by magnetometer measurement update essentially arise from the disagreements between the accelerometer and magnetometer outputs, which can also be caused by non-gravitational acceleration and accelerometer measurement errors. The improved algorithm prevents the magnetometer output from modifying the attitude estimation because firstly the gravity resolved in the body frame can provide sufficient attitude information, and secondly the accelerometer output is assumed more reliable than the magnetometer output in magnetically disturbed environments as has been accepted and verified in many applications.

#### 4.2.4. Yaw Estimation

**Figure 11:**
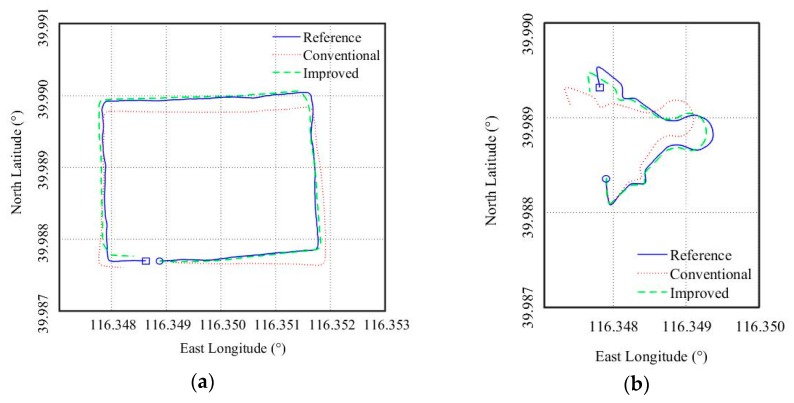
Dead reckoning results. The circle represents the start point, and the square represents the end point. (**a**) Trajectory A. (**b**) Trajectory B.

We tested two trajectories, which are referred to as A and B. MARG sensor outputs corresponding to the trajectories were processed. To show the performances of the yaw estimation intuitively, we used dead reckoning [29] to reproduce the test trajectories based on the yaw estimations and the vehicle velocity data provided by the GPS unit. The results are shown in Figure 11, where the reference trajectories recorded by the GPS unit is also plotted. Clearly, the reproduced trajectories from the improved algorithm are closer to the reference than that from the conventional algorithm.

The root mean square (RMS) errors of the improved algorithm in trajectory A and B are respectively 1.8° and 2.9°. The reason the improved algorithm performed better in A is, as pointed out earlier, the gyroscope measurement only work effectively in running straight status. A was approximately rectangular and has longer straight roads, whereas B consisted of more turning road with different angles and curvature. Obviously, A was more suitable for the gyroscope measurement to take effect. It should be noted that vehicle status cannot affect the work of the two-step measurement update method, which is also helpful for the yaw estimation.

The RMS errors of the yaw and attitude estimation are listed in Table 2. In addition, we used the Kalman filter with vector selection [7] and the complementary filter with varying gains [21], referred to as VSKF and VGCF respectively, to process the experiment data, and the RMS errors are also listed in Table 2. Clearly, the improved algorithm performed best in the yaw and attitude estimation. In fact, as pointed out in the introduction, the VSKF and VGCF essentially use the same strategy as the conventional algorithm to handle magnetic disturbances: reducing the confidence in the magnetometer measurements, which will result in poor correction for the yaw estimation. Whereas the improved algorithm not only reduce the confidence in the disturbed measurements but also introduce new measurements based on vehicle status, which provide supplemental information for the yaw estimation. In addition, the two-step measurement update method eliminates the impacts of magnetometer errors and magnetic disturbances on attitude estimation, which is also helpful for the yaw estimation.

Note that the better yaw estimation accuracy of the improved algorithm is due to its enhanced ability to deal with magnetic disturbances; and it will have the same level of yaw estimation accuracy as the conventional algorithm in magnetically homogeneous environments.

## 5. Conclusions

An improved yaw estimation algorithm for land vehicle using a MARG sensor was proposed in this paper. Under running straight assumption, we derived the gyroscope measurement equation, which update the gyroscope bias and thus constrain the yaw estimation from changing. The validity of the gyroscope measurement depends on the vehicle status; to determine the vehicle status, we analyzed the correlations between different sensors based on the vehicle kinematic model and Coriolis theorem, and constructed a vehicle status marker used to adjust the weight of the gyroscope measurement. In addition, we designed a two-step measurement update method, which implements the magnetometer measurement update separately and eliminates its impact on attitude estimation. Adopting the gyroscope measurement update and the two-step measurement update method, we improved the conventional yaw estimation algorithm. The improved algorithm was tested in experiments and compared against the conventional algorithm. Based on the experiment results, the performances and characters of the improved algorithm were discussed and the conclusion is as follows. The gyroscope measurement update is an effective supplement to the magnetometer measurement update in magnetically disturbed environments; the two-step measurement update methods make attitude estimation immune to the errors induced by magnetometer measurement update; and the improved algorithm provides more reliable yaw estimation for land vehicles than the conventional algorithm. Finally, it should be noted that the vehicle status marker is based on statistic characteristics between different sensors, which make it robust to disturbances, but on the other hand, insensitive to the varying status. The improvements of its real-time performance and ability to detect the turning status with small angular rates will be the topics of further work.

## Figures and Tables

**Table 1:** RMS errors of the yaw estimation corresponding to different b.

Result	b = 0.005 rad/s	b = 0.01 rad/s	b = 0.015 rad/s
**bx**	6.8°	7.6°	7.9°
**by**	7.1°	8.3°	9.1°
**bz**	11.2°	19.4°	29.0°

**Table 2:** RMS errors of the yaw and attitude estimation.

Algorithm	Yaw (°)	Pitch (°)	Roll (°)
VSKF	4.9	4.0	3.1
VGCF	5.6	3.5	3.3
Conventional	5.2	3.7	2.9
Improved	2.1	1.8	1.6
